# BEZ235 Increases the Sensitivity of Hepatocellular Carcinoma to Sorafenib by Inhibiting PI3K/AKT/mTOR and Inducing Autophagy

**DOI:** 10.1155/2021/5556306

**Published:** 2021-04-16

**Authors:** Weiya Cao, Xueke Liu, Yinci Zhang, Amin Li, Yinghai Xie, Shuping Zhou, Li Song, Ruyue Xu, Yongfang Ma, Shiyu Cai, Xiaolong Tang

**Affiliations:** ^1^Medical School, Anhui University of Science & Technology, Huainan 232001, China; ^2^Institute of Environmentally Friendly Materials and Occupational Health, Anhui University of Science and Technology, Wuhu 241000, China; ^3^Department of Laboratory Medicine, The Fourth Affiliated Hospital, Zhejiang University School of Medicine, Yiwu 322000, China; ^4^First Affiliated Hospital of Medical College, Anhui University of Science & Technology, Huainan 232001, China

## Abstract

Acquired resistance of hepatocellular carcinoma (HCC) to sorafenib (SFB) is the main reason for the failure of SFB treatment of the cancer. Abnormal activation of the PI3K/AKT/mTOR pathway is important in the acquired resistance of SFB. Therefore, we investigated whether BEZ235 (BEZ) could reverse acquired sorafenib resistance by targeting the PI3K/mTOR pathway. A sorafenib-resistant HCC cell line Huh7^R^ was established. MTT assay, clone formation assay, flow cytometry, and immunofluorescence were used to analyze the effects of BEZ235 alone or combined with sorafenib on cell proliferation, cell cycle, apoptosis, and autophagy of Huh7 and Huh7^R^ cells. The antitumor effect was evaluated in animal models of Huh7^R^ xenografts *in vivo*. Western blot was used to detect protein levels of the PI3K/AKT/mTOR pathway and related effector molecules. *In vitro* results showed that the Huh7^R^ had a stronger proliferation ability and antiapoptosis effect than did Huh7, and sorafenib had no inhibitory effect on Huh7^R^. SFB + BEZ inhibited the activation of the PI3K/AKT/mTOR pathway caused by sorafenib. Moreover, SFB + BEZ inhibited the proliferation and cloning ability, blocked the cell cycle in the G0/G1 phase, and promoted apoptosis in the two cell lines. The autophagy level in Huh7^R^ cells was higher than in Huh7 cells, and BEZ or SFB + BEZ further promoted autophagy in the two cell lines. *In vivo*, SFB + BEZ inhibited tumor growth by inducing apoptosis and autophagy. We concluded that BEZ235 enhanced the sensitivity of sorafenib through suppressing the PI3K/AKT/mTOR pathway and inducing autophagy. These observations may provide the experimental basis for sorafenib combined with BEZ235 in trial treatment of HCC.

## 1. Introduction

Hepatocellular carcinoma (HCC) is one of the commonest malignancies in the world and the second leading cause of cancer-related death [1–3]. However, the response of advanced HCC to current clinical treatment is poor, mainly because of resistance to chemotherapeutic agents [4]. Therefore, the mechanism of HCC drug resistance and strategies to prevent or reverse the resistance deserves investigation.

Sorafenib, a broad-spectrum tyrosinase inhibitor, reduces angiogenesis and induces apoptosis in tumor cells by inhibiting Raf/MEK/ERK. Sorafenib is the only drug approved by the U.S. Food and Drug Administration for the treatment of patients with advanced HCC [5, 6]. However, owing both to the biological heterogeneity of HCC and the broad action of sorafenib, acquired resistance to sorafenib is a major contributor to the failure of sorafenib treatment in advanced HCC patients [7–9].

Drug resistance refers to the tumor's resistance to chemotherapy drugs [10, 11]. Once drug resistance occurs, its therapeutic effect is substantially reduced. The time of expression of drug resistance varies among individuals [12]. In the treatment of liver cancer, the average time of resistance to sorafenib is one year, but it may occur as early as 3-4 months or only 1-2 years later.

Many mechanisms are involved in the resistance of HCC to sorafenib, of which the PI3K/AKT/mTOR pathway plays an important role [12]. Activation of the pathway is key to cell survival and is closely related to the occurrence of many kinds of tumors [13]. Activated AKT increases the tolerance of tumor cells to apoptosis and promotes abnormal cell growth and metabolism. p-mTOR causes rapid proliferation of tumor cells, increased secretion of oncoproteins, acceleration of the cell cycle, and shortening of the G1 phase. Overactivation of PI3K/AKT/mTOR has been proposed as a mechanism for resistance of HCC to sorafenib, and mTOR inhibitor causing feedback activation of AKT has been reported in some cases [12]. Therefore, the administration of a combination of a PI3K and mTOR dual inhibitor and sorafenib is theoretically appealing for treatment of HCC.

NVP-BEZ235 is a selective dual inhibitor of PI3K and mTOR, which reversibly inhibits the catalytic activity of these kinases through the competitive ATP binding site [14, 15]. *In vitro*, BEZ235 strongly inhibits tumor-cell proliferation and induces cell-cycle arrest in the G1 phase [16]. Oral administration of BEZ235 has a good anticancer effect on nude mouse xenograft models, without significant side effects [17]. BEZ235 also has had a good therapeutic effect against breast cancer, prostate cancer, lung cancer, and myeloid leukemia [18–21].

In this study, we investigated the possible causes of resistance to sorafenib in Huh7^R^ cells and the mechanism of the antitumor effect of sorafenib combined with BEZ235. Our findings suggest that BEZ235 enhances antitumor activity of sorafenib in HCC cells by inhibiting PI3K/AKT/mTOR and inducing autophagy. The combination of BEZ235 and sorafenib might be a promising therapeutic strategy for the treatment of HCC.

## 2. Material and Methods

### 2.1. Cell Lines and Culture

Huh7 cell line was purchased from American type culture collection (Rockville, MD, USA). The sorafenib-resistant hepatoma cell line Huh7^R^ was induced and constructed in our laboratory. Cells were cultured in RPMI-1640 medium containing 15% fetal bovine serum at 37°C, 5% CO_2_.

### 2.2. Construction of Sorafenib-Resistant Cell Line

Huh7 cells in the logarithmic growth phase were selected and cultured at a drug concentration lower than IC_50_ and continuously cultured with a 0.5 *μ*M increasing concentration gradient method for passage. When the drug concentration was increased to 20 *μ*M in stable culture, proliferation and apoptosis of induced cells and sensitive cells were assessed to confirm successful construction of the cell line and determine whether there were differences in the effects of sorafenib on the two kinds of cells. The constructed cell line was cryopreserved for use in subsequent experiments.

### 2.3. Reagents and Antibodies

3-(4,5-Dimethyl-2-thiazolyl)-2,5-diphenyl-2-Htetrazoli*μ*M bromide (MTT) and Annexin V-FITC/PI Kit were purchased from Sigma-Aldrich (St. Louis, MO, USA); PI3K Antibody Kit (9655#), p-Akt Antibody Kit (9916#), mTOR Antibody Kit (9964#), Apoptosis Antibody Kit (9915#), Autophagy Sampler Kit(#4445 T), ULK1 Substrate Ab Sampler Kit (85493 T), and secondary goat anti-rabbit/anti-mouse antibodies were purchased from Cell Signal Technology (Danvers, MA, USA); antibodies to phospho-Rb, cyclin D1, and GSK3*β* were purchased from Abcam Biological Technology (USA), and antibody to *β*-actin and BCA-200 protein assay kit was obtained from Biosharp life science (China). Phosphatase inhibitor tablets were purchased from Beyotime Biotechnology (China). Protein from cell lysates was biotinylated with Antibody Array Assay Kit (Shanghai Biochip Co., Ltd., Shanghai, China). Sorafenib (8705S#, CST, USA), chloroquine (14774#, CST, USA), and BEZ235 (MedChem Express, Dactolisib, USA) were dissolved in dimethyl sulfoxide (DMSO) to produce 10 mM stock solution and stored at -20°C.

### 2.4. MTT Assay

The cell suspensions were diluted to 4 × 10^4^ cells/ml, inoculated on 96-well plates (100 *μ*L/well), and cultured in 5% CO_2_, 37°C, in a constant-temperature incubator overnight. Concentrations of 0.5, 1, 2, 4, 8, 16, and 32 *μ*M sorafenib-treated cells were incubated for 24, 48, and 72 h. Ten microliters of MTT (5 mg/ml) were added to each well and incubated for 4 h. One hundred microliters of DMSO were added to each well and shaken for 10 min. Absorbance was measured at 490 nm, and IC_50_ was calculated. Cells were treated with concentrations of sorafenib below IC_50_ combined with concentrations of BEZ235 (0.05, 0.1, 0.2, 0.4, 0.8, 1.6, and 3.2 *μ*M) for 24 h. The absorbance of each group was detected with the same method, and the inhibitory effects of BEZ235 and sorafenib on Huh7 and Huh7^R^ cells were calculated according to the formula: Inhibition rate (%) = (control group − experimental group)/(control group − blank group) × 100%.

### 2.5. Colony-Formation Assay

Huh7 and Huh7^R^ cells were cultured in 6-well plates at a density of 1000 cells/well overnight. The cells were then treated with sorafenib (1 *μ*M), BEZ (0.1 *μ*M), and SFB (1 *μ*M) + BEZ (+0.1 *μ*M) for about two weeks. When the cell clones were visible, they were washed lightly with PBS twice and fixed with 4% paraformaldehyde for 15 min at room temperature. The cells were washed lightly with PBS twice again and stained with 0.1% crystal violet for 30 min. The cells were washed with PBS to remove the dye solution and dried. Photographs were taken, and the number of colonies was enumerated.

### 2.6. Cell Scratch Healing Test

Huh7 and Huh7^R^ cells (5 × 10^5^ cells/well) were plated overnight in 6-well plates. A cell-free zone on the cell monolayer was made with a sterile 10 *μ*L tip. The cells were continually cultured in a serum-free medium containing drugs. The treatment groups were photographed at 0 h, 24 h, and 48 h. Cell migration ability of the treatment groups was evaluated with the scratch-healing size compared with that of the control group.

### 2.7. Cell-Cycle Analysis

Huh7 and Huh7^R^ cells at 6 × 10^6^ cells/well were incubated in 6-well plates overnight, and various drug combinations were added for 24 h. The RPMI-1640 medium containing the drugs was discarded, and the cells were washed 3 times with PBS. The cells were treated with 0.25% trypsin EDTA, collected into 10 mL centrifuge tubes, and centrifuged at 1000 rpm for 5 min. The cells were resuspended 3 times in PBS, fixed with 700 *μ*L of 75% alcohol for 1 h, and lightly washed 3 times with PBS again. Each treatment group was reacted with 100 *μ*l RNaseA (100 ng/ml) and 500 *μ*l propidium iodide (50 ng/ml) for 30 min at room temperature in the dark. Cells were detected by flow cytometry (BD FASC Calibur, USA) within 1 h.

### 2.8. Apoptosis Detection

Huh7 and Huh7^R^ cells were incubated in 6-well plates at 6 × 10^6^ cells/well overnight. The various drug groups were added for 24 h. The RPMI-1640 medium containing the drugs was discarded, and the cells were washed 3 times with PBS. The cells were treated with 0.25% trypsin EDTA, collected into a 10 mL centrifuge tube, and centrifuged at 1000 rpm for 5 min. The cells were resuspended 3 times in PBS. Each treatment group was stain with 5 *μ*L Annexin V-FITC and 10 *μ*l propidium iodide at room temperature in the dark for 10-15 min. Cells were detected by flow cytometry (BD FASC Calibur, USA) within 1 h.

### 2.9. Indirect Immunofluorescence

Cells (2.5 × 10^5^ cells/well) were incubated overnight in 24-well plates containing cell slides. Cells were treated with drugs for 24 h and fixed with 4% paraformaldehyde for 15 min at room temperature. LC3 primary antibody (LC3A/B (D3U4C) XP® Rabbit mAb, 12741#, CST, USA) was incubated overnight at 4°C. Cells were lightly washed 3 times with PBS, incubated with an Alexa Fluor 488® conjugated second antibody (anti-Rabbit IgG (H + L), F(ab')_2_ Fragment, 4412#, CST, USA) for 1 h, and counterstained with Hoechst 33258 (Thermo Fisher Scientific). Cells were visualized with a fluorescence microscope.

### 2.10. Western Blot Analysis

Protein was extracted from the cells with a mixture of radio immunoprecipitation assay and protease inhibitor (Beyotime Biotechnology, Shanghai, China). The protein concentration was measured with a BCA200 protein assay kit (Biosharp Life Science, Hefei, China). SDS-polyacrylamide gel electrophoresis (SDS-PAGE) was carried out, and proteins were electrotransferred onto a polyvinylidene fluoride membrane (Millipore, USA). Membranes were blocked with 5% skim milk for 1 h, incubated with primary antibody overnight at 4°C, and incubated with second antibody at room temperature for 1 h. Protein bands were visualized with an ECL detection kit (Thermo Fisher Scientific Waltham, MA, USA), captured with a gel Bio-Rad (Hercules, CA, USA) scanning, and quantified with Image J Version 1.48 software (NIH, Bethesda, MD).

### 2.11. Tumor Xenograft Experiments

Female BALB/c nude mice (Vital River Laboratories, Beijing, China) were used for animal studies. The mice were housed and maintained in the SPF-level animal room of the Central Laboratory of Medical School Anhui University of Science and Technology, Anhui, People's Republic of China (no: AUST2018-10088). All animal studies were carried out in accordance with ARRIVE guidelines [22] and approved by the Animal Experimental Ethics Committee of Anhui University of Science and Technology.

The mice were injected subcutaneously with 100 *μ*L of Huh7^R^ cell suspension (2 × 10^7^/mL) into the dorsal right side. When tumors were about 100 mm^3^, mice were randomized into 4 groups: control group, 100 *μ*L saline daily, ip; SFB group, 30 mg/kg daily, orally; BEZ235 group, 45 mg/kg daily orally; and sorafenib + BEZ235 (SFB + BEZ) group, SFB (30 mg/kg daily orally plus BEZ235 (45 mg/kg daily orally). The dose of BEZ235 and sorafenib used in vivo was based on recommended dosage according to drug instructions. Each group of mice received treatment for 4 weeks, and tumor size and body weight were measured every 3 days. Tumor volume was calculated according to the equation: length × (width)^2^)/2. Mice were sacrificed after treatment, tumor tissues were isolated, and protein was extracted for western blot detection.

### 2.12. Statistical Analysis

All data were analyzed with IBM SPSS Statistics 24.0. Data were presented as mean ± SD. All experiments were performed in triplicate. Group differences were calculated with *t* test or one-way ANOVA. Significance was defined as *p* < 0.05. ImageJ 1.44p software (http://imagej.nih.gov/ij/) was used for quantitative analysis of protein immunoblotting.

## 3. Results

### 3.1. Validation of Huh7^R^ Strains

**Figure 1:**
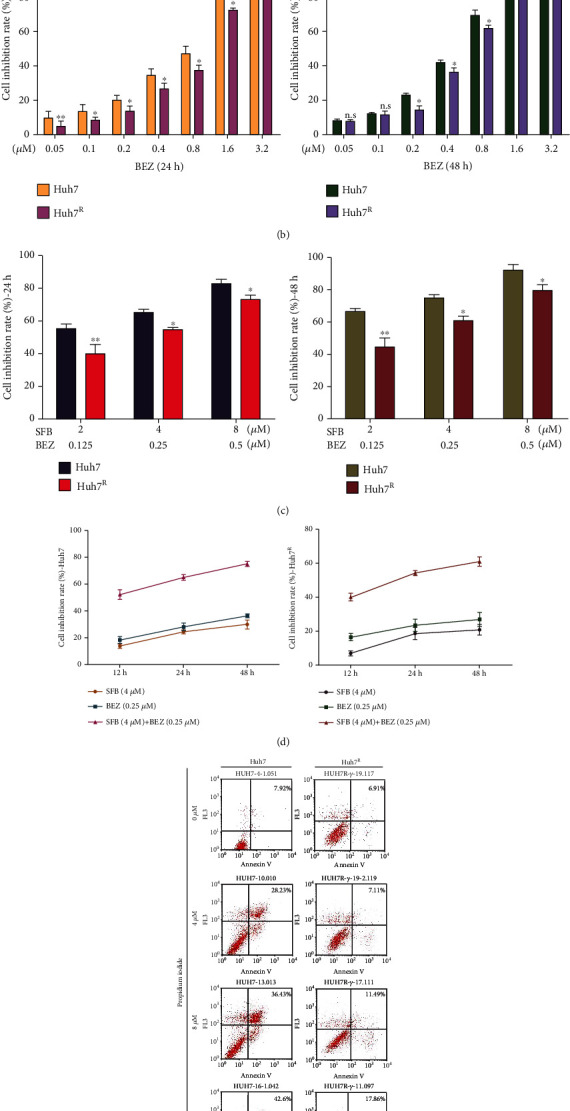
Cell viability of Huh7 and Huh7^R^ cells and the effect of SFB on apoptosis in Huh7 and Huh7^R^ cells. (a and b) The inhibitory effect (IC_50_) of sorafenib or BEZ235 on Huh7 and Huh7^R^ at various concentrations for 24 or 48 h. (c) The inhibition rate of proliferation in each group at various concentrations of sorafenib. (d) The effect of sorafenib, BEZ235, or sorafenib combined with BEZ235 on cell proliferation at 12, 24, and 48 h (SFB, 4 *μ*M; BEZ235, 0.25 *μ*M; SFB 4 *μ*M + BEZ 0.25 *μ*M). (e) The effect of sorafenib on apoptosis in Huh7 and Huh7^R^ cells. Data are presented as mean ± SD, *n* = 3. ^∗^*p* < 0.05, ^∗∗^*p* < 0.01, ^∗∗∗^*p* < 0.001 all versus SFB group. SFB: sorafenib; BEZ: BEZ235; SFB + BEZ: sorafenib combined with BEZ235.

After more than 6 months of continuous pressure with sorafenib on Huh7, resistant strains (Huh7^R^) were produced. To verify the resistance of Huh7^R^, cell proliferation and apoptosis were measured with MTT and flow cytometry, respectively. Huh7 and Huh7^R^ cells were treated with sorafenib (0.5, 1, 2, 4, 8, 16, and 32 *μ*M) for 24, 48, and 72 h. The result showed that the IC_50_ (*μ*M) of Huh7 and Huh7^R^ was 13.5 and 24.7 at 24 h; 8.67 and 17.0 at 48 h; and 6.85 and 11.1 at 72 h (data not shown), respectively (Figures 1(a) and 1(b)). Annexia V-positive and propidium iodide-negative cells are considered early apoptotic cells. The results of Annexin V/propidium iodide double staining revealed that under the action of sorafenib 0, 4, 8, or 16 *μ*M for 24 h, the apoptosis rate of Huh7 was 8.52%, 28.2%, 36.4%, and 41.6% (Figure 1(e)), and that of Huh7^R^ was 6.91%, 7.11%, 10.3%, and 17.9% (Figure 1(e)). The differences between the groups were statistically significant (*p* < 0.01) and indicated that Huh7^R^ had been produced.

### 3.2. BEZ235 Increases the Inhibitory Effect of Sorafenib on Cell Proliferation and Migration

To verify the inhibitory effect of sorafenib combined with BEZ235 on the viability of Huh7^R^ cells, we first treated Huh7 and Huh7^R^ cells with various concentrations of BEZ235 (0.05, 0.1, 0.2, 0.4, 0.8, 1.6, and 3.2 *μ*M) for 24 h and 48 h to determine their IC_50_. The results showed that the IC_50_ (*μ*M) of BEZ235 in Huh7 and Huh7^R^ was 0.705 and 0.806 at 24 h, and 0.466 and 0.62 at 48 h, respectively (Figure 1(b)). The combination of sorafenib and BEZ235 at various concentrations inhibited the proliferation of Huh7 and Huh7^R^ in a concentration-dependent manner (Figure 1(c)), and SFB + BEZ exhibited stronger inhibition than did either agent alone (Figure 1(d)).

Sorafenib (4 *μ*M) exerted a significant inhibitory effect, and the value below IC_50_ was suitable for subsequent experiments. The effect of combined 0.25 *μ*M BEZ235 and sorafenib was stronger than that of 0.05 *μ*M and was like that of the effect of 0.5 *μ*M. Therefore, we chose 0.25 *μ*M as the suitable concentration for these experiments. Because too long incubation time may cause other toxicities to the cells, we sought to determine an appropriate incubation time; exposure for 24 h inhibited Huh7^R^ proliferation and promoted cell apoptosis. Therefore, we chose a combination of sorafenib (4 *μ*M) and BEZ235 (0.25 *μ*M) and incubation for 24 h to estimate the effect of the two-drug combination.

**Figure 2:**
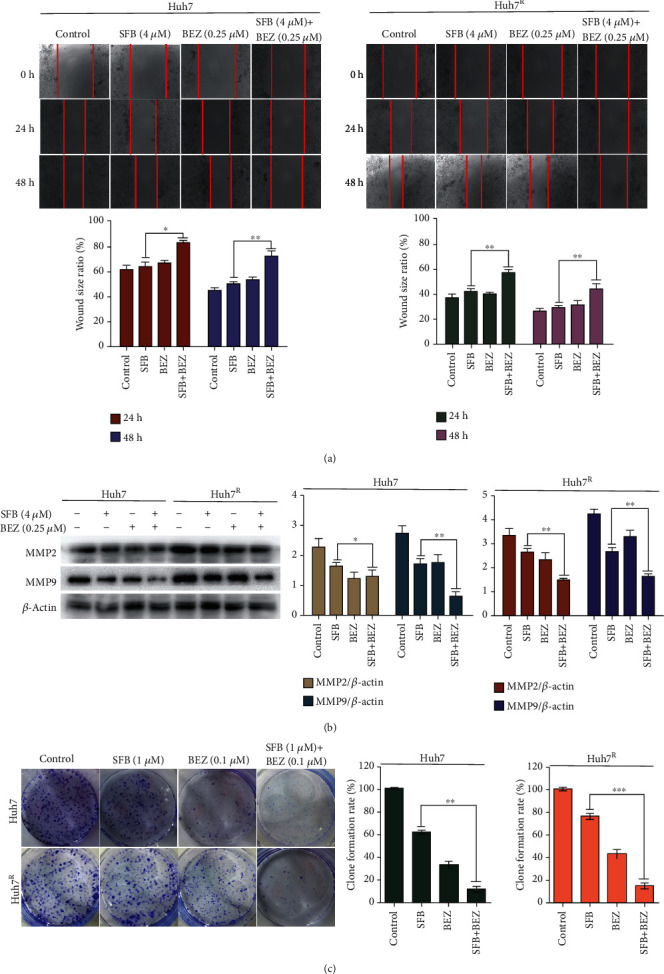
Effects of sorafenib combined with BEZ235 on Huh7 or Huh7^R^ cell migration and colony formation. (a) Degree of scratch healing at 0, 24, and 48 h in each group. (SFB, 4 *μ*M; BEZ235, 0.25 *μ*M; and SFB, 4 *μ*M + BEZ, 0.25 *μ*M). ×100 magnification. (b) Huh7 or Huh7^R^ cells were incubated with various treatments for 24 h (SFB, 4 *μ*M; BEZ235, 0.25 *μ*M; and SFB, 4 *μ*M + BEZ, 0.25 *μ*M). The cell lysates were collected, and the designated proteins (MMP2, MMP9) were detected by western blot. (c) Colony formation assay was carried out to assess the proliferation of Huh7 and Huh7^R^ cells for 24 h (control; SFB, 1 *μ*M; BEZ, 0.1 *μ*M; and SFB 1 *μ*M + BEZ 0.1 *μ*M). Data are presented as mean ± SD, *n* = 3. ^∗^*p* < 0.05, ^∗∗^*p* < 0.01 all versus SFB single agent. SFB: sorafenib; BEZ: BEZ235; SFB + BEZ: sorafenib combined with BEZ235.

We next evaluated the effects of various treatments on cell migration through scratch healing experiments. As shown in Figure 2(a), compared with the control group, the SFB or BEZ prevented the migration of Huh7 and Huh7^R^, and compared with SFB monotherapy, SFB + BEZ had more inhibition. The expression of migration protein (MMP2, MMP9) had the same trend (Figure 2(b)); that is, SFB + BEZ had a stronger migration inhibitory effect than did SFB alone. These results are evidence that BEZ235 reduces the resistance of hepatoma cells induced by sorafenib and increases the sensitivity to the drug, leading to inhibition of cell growth and promotion of cell death. We performed colony-formation assays in Huh7 and Huh7^R^ to evaluate the effect of various treatments. As illustrated in Figure 2(c), sorafenib single therapy did not significantly decrease Huh7^R^ cell colony formation compared with the control values, whereas SFB + BEZ has more inhibitory effect than did SFB alone on Huh7 or Huh7^R^.

### 3.3. Sorafenib Combined with BEZ235 Promoted G0/G1 Cycle Arrest in Huh7 and Huh7^R^ Cells

**Figure 3:**
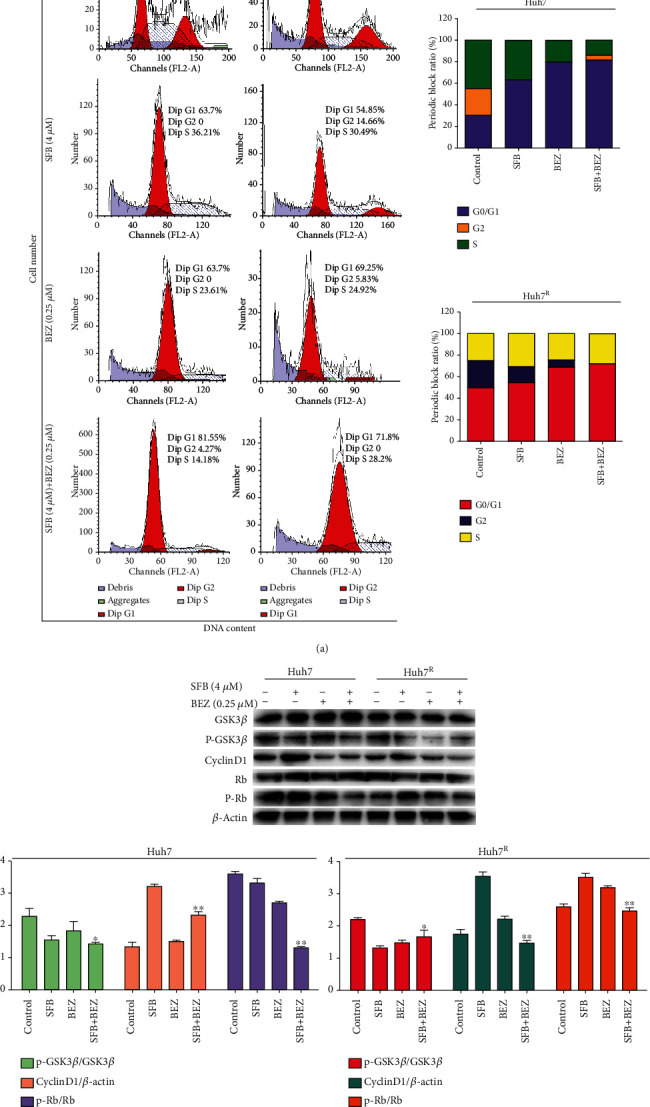
The effect of various treatments on the cell cycle in Huh7 or Huh7^R^, as determined with flow cytometry and western blot. (a) Huh7 or Huh7^R^ cells stained with propidium iodide after treatment for 24 h. (SFB, 4 *μ*M; BEZ235, 0.25 *μ*M; SFB, 4 *μ*M + BEZ, 0.25 *μ*M). Cell-cycle distribution was analyzed by flow cytometry. (b) Western blot analysis revealed the expression of the key G1-phase regulatory protein after treatment for 24 h: cyclin-D1 and p-Rb and p-GSK3*β*. (SFB, 4 *μ*M; BEZ235, 0.25 *μ*M; and SFB, 4 *μ*M + BEZ, + 0.25 *μ*M). Data are presented as mean ± SD, *n* = 3. ^∗^*p* < 0.05, ^∗∗^*p* < 0.01 all versus SFB single agent. SFB: sorafenib; BEZ: BEZ235; SFB + BEZ: sorafenib combined with BEZ235.

To investigate whether the inhibitory effect of sorafenib combined with BEZ235 on cell proliferation is related to cell-cycle disruption, we used propidium iodide staining and flow cytometry to analyze the cell cycle in Huh7 and Huh7^R^ treated by SFB (4 *μ*M), BEZ (0.25 *μ*M), or SFB (4 *μ*M) plus BEZ (0.25 *μ*M) for 24 h. The G0/G1 phase cell number percentages of SFB- or BEZ-treated cells (Figure 3(a)) in Huh7 were 63.8% and 79.6% and in Huh7^R^ were 54.9% and 71.8%, respectively; the G0/G1 phase cell percentages of G0/G1 phase of SFB + BEZ in Huh7 and Huh7^R^ were 81.6% and 69.3%, respectively. These results are evidence that BEZ235 enhances the G0/G1 phase arrest induced by sorafenib.

The proteins cyclin D1 and p-Rb govern G1 to S phase progression. Thus, we examined the expression of endogenous cyclin D1, p-Rb, and their upstream regulatory protein GSK3*β*. As indicated in Figure 3(b), in Huh7 and Huh7^R^, compared with sorafenib treatment alone, p-GSK3*β*, cyclin D1, and p-Rb values were lower in cells treated with SFB + BEZ. These data are evidence that BEZ235 enhances sorafenib-induced cell-cycle arrest at G0/G1 through the downregulation of p-GSK3*β* and the key G1 phase regulatory proteins cyclin D1 and p-Rb.

### 3.4. BEZ235 Enhanced Sorafenib Induced Apoptosis in Huh7 and Huh7^R^ Cells

**Figure 4:**
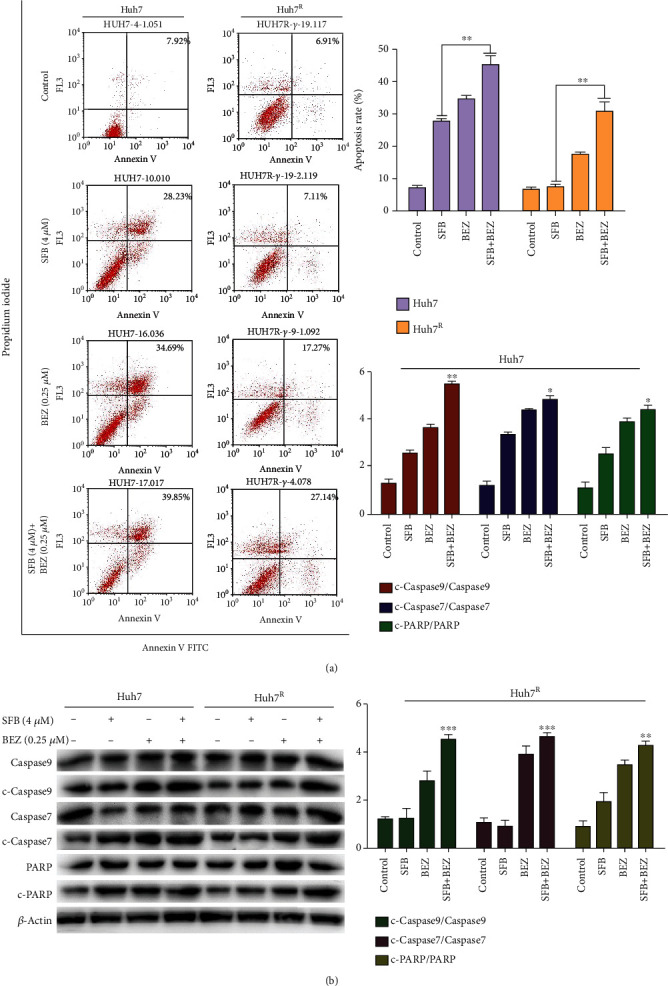
The effect of SFB + BEZ on apoptosis in Huh7 and Huh7^R^ cells. (a) Apoptosis rate as determined with Annexin V FITC/PI staining after treatment with various concentrations of sorafenib for 24 h and observed with flow cytometry. ×400 magnification. (b) Huh7 and Huh7^R^ cells were treated for 24 h and observed with flow cytometry (SFB, 4 *μ*M; BEZ235, 0.25 *μ*M; and SFB, 4 *μ*M + BEZ, + 0.25 *μ*M). ×400 magnification. (c) The expression levels of caspase proteins (caspase 9, caspase 7, and PARP) in each treatment group (24 h) were analyzed by western blot. The ratios of apoptosis-related proteins to total protein were determined. Data are presented as mean ± SD, *n* = 3. ^∗^*p* < 0.05, ^∗∗^*p* < 0.01 all versus SFB single-agent group. SFB: sorafenib; BEZ: BEZ235; SFB + BEZ: sorafenib combined with BEZ23. c-caspase 9: cleaved-caspase 9; c-caspase 7: cleaved-caspase 7; c-PARP: cleaved-PARP.

We next studied the effect of sorafenib on apoptosis in HCC cells with Annexin V FITC/PI staining after various treatments for 24 h. In Huh7, the apoptosis rate of SFB + BEZ (39.9%) was higher than that of SFB (28.2%); in Huh7^R^, the apoptosis rate of SFB + BEZ (27.1%) was significantly higher than that of SFB (7.11%), suggesting that BEZ235 enhances sorafenib-induced apoptosis in both Huh7 and Huh7^R^ cells (Figure 4(a)).

To verify the mechanisms of sorafenib and BEZ235 on apoptosis in Huh7 and Huh7^R^, western blot was used to detect the expression levels of apoptosis-related proteins PARP, caspase 9, and caspase 7. The results showed that the levels of c-PARP, cleaved-caspase 7, and cleaved-caspase 9 in Huh7 were higher than in Huh7^R^ in the SFB (4 *μ*M) group (Figure 4(b)). These results indicated that part of the apoptosis activation of Huh7^R^ was inhibited. However, the expression of c-PARP, cleaved caspase7, and cleaved caspase 9 in SFB + BEZ-treated cells was significantly than in cells treated with SFB alone. These results indicated that BEZ235 largely restored the sensitivity of Huh7^R^ to sorafenib.

### 3.5. BEZ235 Enhances the Autophagy Effect Induced by Sorafenib in Huh7 and Huh7^R^ Cells

**Figure 5:**
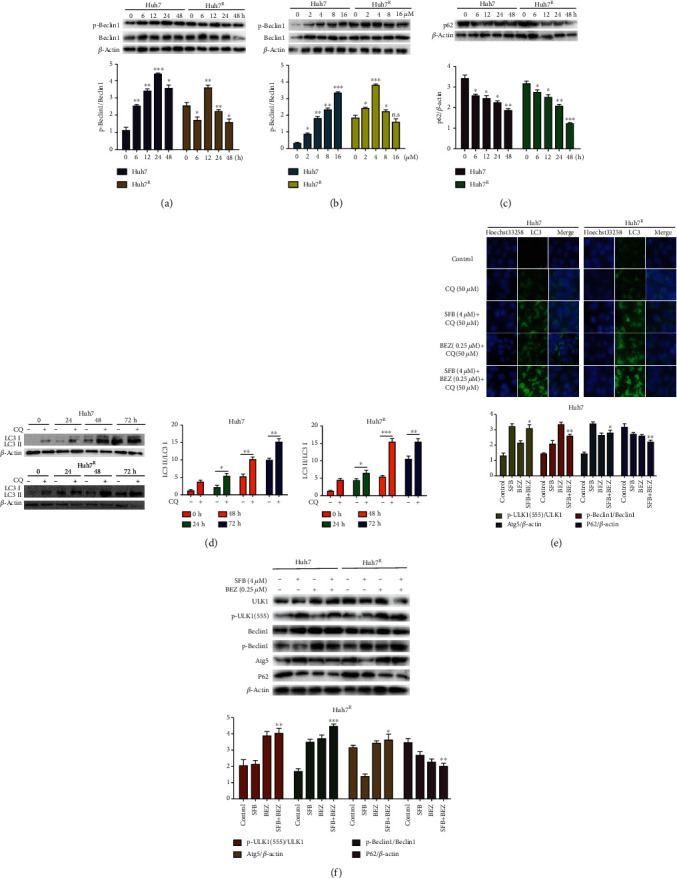
Effect of BEZ235 on sorafenib-induced autophagy in HCC cells. (a and c) The autophagy-related proteins (Beclin1, p62) were estimated by western blot after treatment with sorafenib (4 *μ*M) for 0, 6, 12, 24, and 48 h. (b) Huh7 and Huh7^R^ cells were treated with sorafenib (2, 4, 8, and 16 *μ*M) for 24 h and observed with western blot. (d) LC3 was estimated by western blot after treatment with sorafenib (4 *μ*M) and chloroquine (50 *μ*M) for 0, 24, 48, and 72 h. (e) Indirect immunofluorescence was used to detect the effect of various treatments on the expression of LC3 after treatment with chloroquine (CQ), sorafenib, BEZ235, or various combinations of them for 24 h. (CQ, 50 *μ*M; SFB + CQ, 4 *μ*M+ 50 *μ*M; BEZ + CQ: 0.25 *μ*M+ 50 *μ*M). (f and g) Huh7 and Huh7^R^ cells were incubated with various treatments for 24 h. The cell lysates were collected, and the designated proteins were detected by western blot (SFB, 4 *μ*M; BEZ235, 0.25 *μ*M; SFB. 4 *μ*M + BEZ, 0.25 *μ*M; CQ, 50 *μ*M). Data are presented as mean ± SD, *n* = 3. ^∗^*p* < 0.05, ^∗∗^*p* < 0.01 all versus SFB or CQ single-agent group. CQ: chloroquine; SFB: sorafenib; BEZ: BEZ235; SFB + BEZ: sorafenib combined with BEZ235.

To test whether there were differences in autophagy with various treatments, we used 4 *μ*M sorafenib to treat Huh7 and Huh7^R^ cells for 6, 12, 24, and 48 h, and 0, 2, 4, 8, and 16 *μ*M sorafenib to treat cells for 24 h, then detected the expression of proteins. In the control group, basic activation of Beclin1, the key molecule in autophagy, was higher in Huh7^R^ than in Huh7, suggesting that there was a higher level of autophagy in Huh7^R^ (Figure 5(a)); In Huh7, p-Beclin1 had a significant time and concentration dependence (Figure 5(a)); compared with Huh7, in Huh7^R^, p-Beclin1 gradually increased with time, peaked at 12 h (Figure 5(a)), and then decreased, suggesting that sorafenib caused a transient increase in Huh7^R^. p-Beclin1 was expressed in a concentration-dependent manner in Huh7 cells, but there was a peak in Huh7^R^ (Figure 5(b)). In Huh7, P62 decreased in a time-dependent manner (Figure 5(c)); in Huh7^R^, p62 decreased gradually (Figure 5(c)). The expression of LC3 was increased in a time-dependent manner in Huh7 (Figure 5(d), D1), while it was increased to a peak at 48 h and then decreased in Huh7^R^ (Figure 5(d), D2). Concentrations of other autophagic molecules, p-ULK1 (555), p-Beclin1, and Atg5, in Huh7^R^ were significantly higher than those in Huh7 after treatment with 4 *μ*M sorafenib (Figure 5(f)). Huh7^R^ cells are not as sensitive to SFB as Huh7 are, perhaps due to drug resistance, and the expression of Atg5 in Huh7 was increased, whereas in Huh7^R^ cells, it was slightly decreased under the treatment of SFB (4 *μ*M). These results indicated that the degree of autophagy in Huh7^R^ was enhanced. The expression of these proteins in the SFB + BEZ group was significantly lower than in the control group and the single-drug group. These results suggest that attenuation of autophagy is one of the reasons for sorafenib resistance in Huh7^R^ cells and that BEZ235 enhances the autophagy effect of sorafenib in Huh7 and Huh7^R^ cells.

We next measured the expression of LC3 in various treatments for 24 h with indirect immunofluorescence. The results revealed that in Huh7 or Huh7^R^, sorafenib or BEZ235 induced a transient increase of autophagy flow (Figure 5(e)). Compared with Huh7, the control group in Huh7^R^ had a certain fluorescence intensity of LC3 (Figure 5(e)); sorafenib and BEZ235 caused the expression of LC3, and the effect of BEZ235 and their combination was greater. Similar results were also reflected in the western blot results (Figure 5(g), G1 and G2).

### 3.6. BEZ235 Antagonizes the Abnormal Activation of PI3K/AKT/mTOR Pathway Induced by Sorafenib in Huh7 and Huh7^R^ Cells

**Figure 6:**
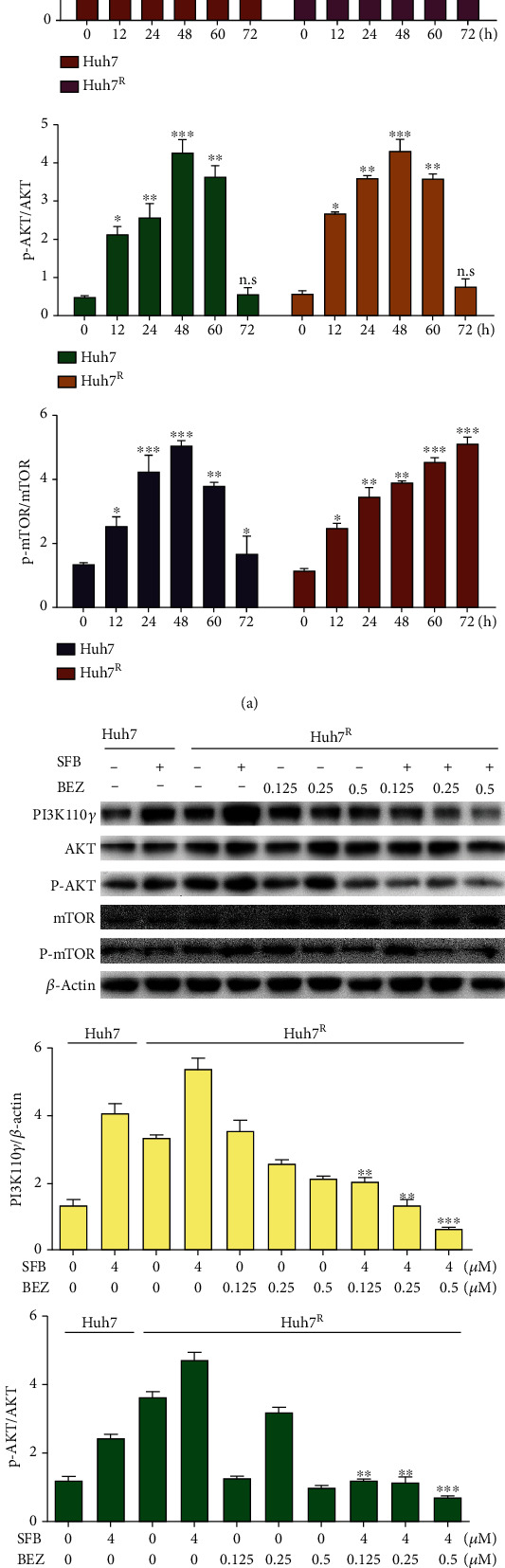
BEZ235 blocked the activation of the PI3K/mTOR pathway induced by sorafenib. (a) Sorafenib (4 *μ*M) activated the PI3K/AKT/mTOR pathway in Huh7 and Huh7^R^ cells. (b) BEZ235 countered the abnormal activation of the PI3K/AKT/mTOR pathway induced by sorafenib. Data are presented as mean ± SD, *n* = 3. ^∗^*p* < 0.05, ^∗∗^*p* < 0.01 all versus SFB single-agent group. SFB: sorafenib; BEZ: BEZ235; SFB + BEZ: sorafenib combined with BEZ235.

The role of PI3K/AKT/mTOR in acquired sorafenib resistance was measured with western blot. Huh7 and Huh7^R^ were treated with sorafenib (4 *μ*M) for 12, 24, 48, 60, or 72 h. As shown in Figure 6(a), sorafenib promoted PI3K/AKT/mTOR phosphorylation in Huh7 or Huh7^R^ cells. In Huh7, the p-AKT and p-mTOR activation levels reached a peak at 48 h, then gradually declined. In Huh7^R^, the phosphorylation occurred from the start of drug addition, maintaining a stable expression level in 24-60 h in p-AKT, but p-mTOR was stably high-expressed for 24-72 h. The activation of PI3K/AKT/mTOR pathway was more in Huh7^R^, and AKT and mTOR had a continuous activation trend.

As shown in Figure 6(b), there was no evident inhibitory effect of SFB (4 *μ*M) + BEZ (0.125 *μ*M) on PI3K/AKT/mTOR, whereas SFB (4 *μ*M) + BEZ (0.25 *μ*M) and SFB (4 *μ*M) + BEZ (0.5 *μ*M) inhibited the abnormal activation of PI3K/AKT/mTOR. Therefore, in the subsequent experiments, SFB (4 *μ*M) + BEZ (0.25 *μ*M) was used as the experimental combination. Protein analysis results showed that the activation level of PI3K/AKT/mTOR in the SFB + BEZ group was less than in the SFB group, and the inhibition effect of medium (0.25 *μ*M) and high (0.5 *μ*M) concentrations was evident.

### 3.7. The Combination of Sorafenib and BEZ235 Inhibited Tumor Proliferation In Vivo

**Figure 7:**
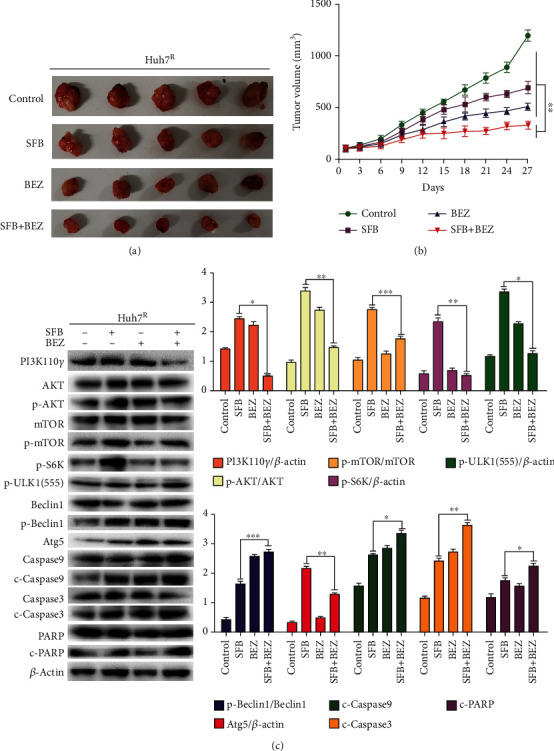
BEZ235 significantly enhanced the antitumor effect of sorafenib in vivo by PI3K/mTOR double inhibition. (a) Tumor tissues of each group were collected after 28-day treatment of tumor-bearing mice. Tumor volume was expressed as mean ± SD. *n* = 5. (b) Antitumor efficacies of sorafenib, BEZ235, and the combination of sorafenib and BEZ235 in Huh7^R^ tumor xenografts. (c) A portion of the tumor tissue was lysed to extract tissue proteins (PI3K110*γ*, p-Akt, p-mTOR, p-ULK1(555), p-Beclin1, Atg5, cleaved caspase 9, cleaved caspase 3, and cleaved PARP) for western blot analysis. Data are presented as mean ± SD, *n* = 3. ^∗^*p* < 0.05, ^∗∗^*p* < 0.01 all versus SFB single-agent group. SFB: sorafenib; BEZ: BEZ235; SFB + BEZ: sorafenib combined with BEZ235.

Antitumor effects of sorafenib combined with BEZ235 *in vivo* were evaluated in a murine xenograft model of Huh7^R^ cells. Tumor tissues of tumor-bearing mice of each group were isolated and collected after 27-day treatment. Tumor growth in animals treated with SFB + BEZ was significantly inhibited compared with that in animals treated with SFB alone (Figures 7(a) and 7(b)). Tissue protein lysates were collected from the Huh7^R^ xenografts and detected by western blot. The results showed that SFB + BEZ significantly reduced the phosphorylation levels of PI3K, AKT, and mTOR and promoted autophagy and apoptosis compared with levels in the control, SFB, and BEZ groups (Figure 7(c)). Thus, we concluded that BEZ235 significantly increased the antitumor effect of sorafenib in HCC *in vivo* at least in part by inhibiting PI3K/Akt/mTOR and inducing the autophagy pathway.

## 4. Discussion

**Figure 8:**
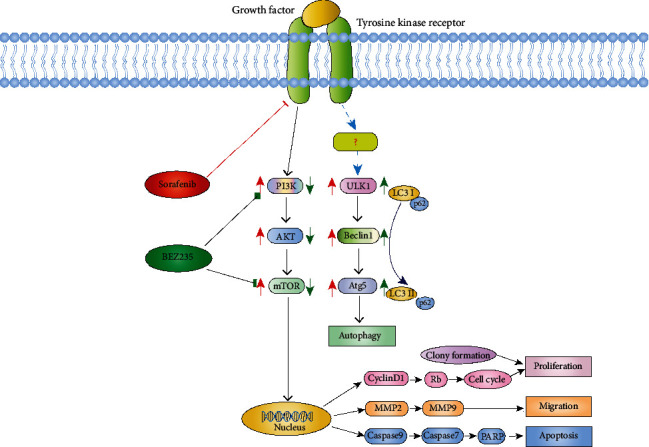
Schematic diagram of the PI3K/AKT/mTOR and autophagy signal pathway. The combination of growth factors and receptors activates the cascade of the PI3K signaling pathway and promotes cell survival, proliferation, and migration. In cell-cycle regulation, GSK3*β* reduces the expression of cyclin D1, thereby inhibiting Rb phosphorylation and blocking the cell cycle. In HCC cells that have chemotherapy resistance to sorafenib, the use of sorafenib can abnormally activate PI3K/AKT/mTOR and induce insignificant autophagy. Sorafenib combined with BEZ235 targets a variety of molecules, inhibits PI3K, and enhances autophagy to inhibit cell proliferation, migration, and cell-cycle progression. Activation of caspase-9 and caspase-7 leads to cell apoptosis.

In this investigation of the possible causes of resistance of HCC to chemotherapeutic agents, the major finding was that BEZ235 enhances antitumor activity of sorafenib in HCC cells by inhibiting PI3K/AKT/mTOR and inducing autophagy (Figure 8). NVP-BEZ235 is a selective dual inhibitor of PI3K and mTOR, which reversibly inhibits the catalytic activity of these kinases through the competitive ATP binding site. The results of this study thus raise the possibility that the combination of BEZ235 and sorafenib can be an effective therapeutic strategy for the treatment of HCC.

HCC is a highly vascularized tumor [23]. According to the research results of the cell culture system and the xenograft model, the abnormal expression and activity of epidermal growth factor receptor (EGFR) [24], platelet-derived growth factor receptor alpha (PDGFR*α*) [25], and insulin-like growth factor (IGF) [26, 27] can regulate PI3K/AKT/mTOR and autophagy pathway. The abnormal expression of these pathways is closely related to the pathogenesis of HCC. We propose that the abnormal expression and activity changes of these growth factor receptors play a certain role in the resistance of HCC to sorafenib. The specific expression and mechanism need to be further explored.

Advanced HCC usually is not sensitive to chemotherapy drugs, so the treatment effect is limited and the median survival period is short [3]. The diversity and dispersion of carcinogenic mutations have made the development of effective anticancer drugs difficult. Compared with the development of other types of tumor-targeting drugs, the development of HCC chemotherapy drugs has lagged.

Sorafenib was approved by the US Food and Drug Administration as a unique target drug for the treatment of advanced HCC in 2007 [28]. With the use of the drug, though, a considerable portion of HCC was found resistant to sorafenib, even multiple-drug resistant [29, 30]. The main strategy for overcoming the resistance to chemotherapy is combination therapy; that is, two drugs that inhibit one or more key signal pathways in different ways, thus, blocking resistance mechanisms [31–33]. There are many mechanisms of acquired sorafenib resistance in HCC cells, of which abnormal activation of the PI3K/AKT/mTOR pathway plays an important role [34].

Changes in sensitivity to apoptosis may be a marker of sorafenib-resistant cells [35]. The apoptosis rate of Huh7^R^ was significantly lower than that of Huh7 treated with sorafenib, which confirmed the construction of Huh7^R^. Sorafenib or BEZ235 caused G0/G1 phase arrest of the cells, and the effect was even more with the two agents combined.

Sorafenib-induced apoptosis of cancer cells is a common mechanism of targeted chemotherapy. There are two established pathways leading to apoptosis: the exogenous pathway of cell death (the pathway of cell-death receptor) and the endogenous pathway of cell death (the pathway of mitochondrial initiation) [36, 37]. Sorafenib induces cancer cell apoptosis mainly through the internal pathway. It has been reported [38] that sorafenib induced apoptosis of several human cancer cell lines by downregulating the level of antiapoptotic proteins. Our results showed that sorafenib combined with BEZ235 significantly increased the amount of apoptosis.

The PI3K/AKT/mTOR pathway is a new target of chemotherapy [39]. In this study, the important node molecules of the PI3K/AKT/mTOR pathway were stress-activated after treatment with sorafenib in Huh7, while they were continuously activated in Huh7^R^. Therefore, Huh7^R^ has stronger survivability. BEZ235, a dual PI3K/mTOR inhibitor, inhibits the PI3K/AKT/mTOR pathway. Therefore, we used BEZ235 combined with sorafenib to explore the therapeutic effect of Huh7 and Huh7^R^. PI3K110*γ*, p-AKT, and p-mTOR of SFB + BEZ group in Huh7^R^ were significantly decreased compared with the SFB group, and the ideal inhibition effect was achieved by the 0.25 *μ*M BEZ235. We speculated that continuous activation of PI3K/AKT/mTOR is responsible for Huh7^R^ resistance, and BEZ235 inhibits the cell survival pathway and increases the sensitivity of Huh7^R^ to sorafenib.

Autophagy, a double-edged sword, is one of the important factors for drug resistance of tumor cells [40, 41]. We found that Huh7^R^ has a high autophagy level, suggesting that the cells could lower the sorafenib concentration and prevent tumor apoptosis through protective autophagy. P62 is a ubiquitin-binding protein and a marker of the autophagy-mediated protein degradation pathway [42, 43]. As an autophagy-specific substrate, it is degraded by the autophagy lysosomal pathway. P62 was decreased in a time-dependent manner in Huh7 and Huh7^R^ and complemented with p-Beclin1. Sorafenib, BEZ235, and their combination could induce autophagy. Similar results were obtained *in vivo.* The SFB + BEZ group showed the highest level, suggesting that autophagy increases the therapeutic effect of chemotherapy drugs on tumors. Therefore, autophagy-mediated acquired sorafenib resistance is a complex mechanism and cannot be generalized.

Patients with advanced HCC need more effective treatments [44]. Animal studies and clinical trials have shown that combination therapy is more effective than single-agent therapy [45, 46]. In this study, we demonstrated the antitumor effect of sorafenib combined with BEZ235. Compared with the Huh7, Huh7^R^ has stronger proliferation and cloning ability and a higher level of autophagy. Importantly, abnormal activation of the PI3K/AKT/mTOR pathway may be the main cause of resistance to sorafenib. Sorafenib plus BEZ235 downregulated the PI3K/AKT/mTOR pathway, reduced cell proliferation, and induced death of Huh7^R^ cells *in vitro*. Finally, we found that SFB + BEZ induced apoptosis and autophagy. There is a question of whether there is an internal connection between the two cell death methods, and the specific mechanism needs further study. Our findings support the strategy of sorafenib and BEZ235 in combination therapy and suggest that the application of an autophagy inducer may increase the efficacy of sorafenib, so as to ensure the effectiveness of cancer treatment.

This study has limitations: it consists mainly of *in vitro* studies of HCC cells, with introductory *in vivo* work: the antitumor effects of sorafenib combined with BEZ235 in a murine xenograft model of Huh7^R^ cells. Nonetheless, we believe that the results have value, and they will lead to our future research that will focus on in-depth *in vivo* studies on the therapeutic effects of BEZ235 in HCC.

## 5. Conclusion

This study established that abnormal activation of the PI3K/AKT/mTOR and autophagy pathway is the main reason for resistance to sorafenib in Huh7 cells. BEZ235 restored the sensitivity of Huh7^R^ to sorafenib and enhanced the antitumor activity of the two drugs by inhibiting these two pathways. These observations can be the foundation for clinical trials evaluating the efficacy of combining sorafenib with BEZ235 for the treatment of HCC.

## Data Availability

The [DATA TYPE] data used to support the findings of this study are included within the article.
